# Supplementary figures and images for: No evidence of fetal defects or anti-syncytin-1 antibody induction following COVID-19 mRNA vaccination
Source: PLoS Biol. 2022 May 24;20(5):e3001506. doi: 10.1371/journal.pbio.3001506 (PMC9129011; doi:10.1371/journal.pbio.3001506)

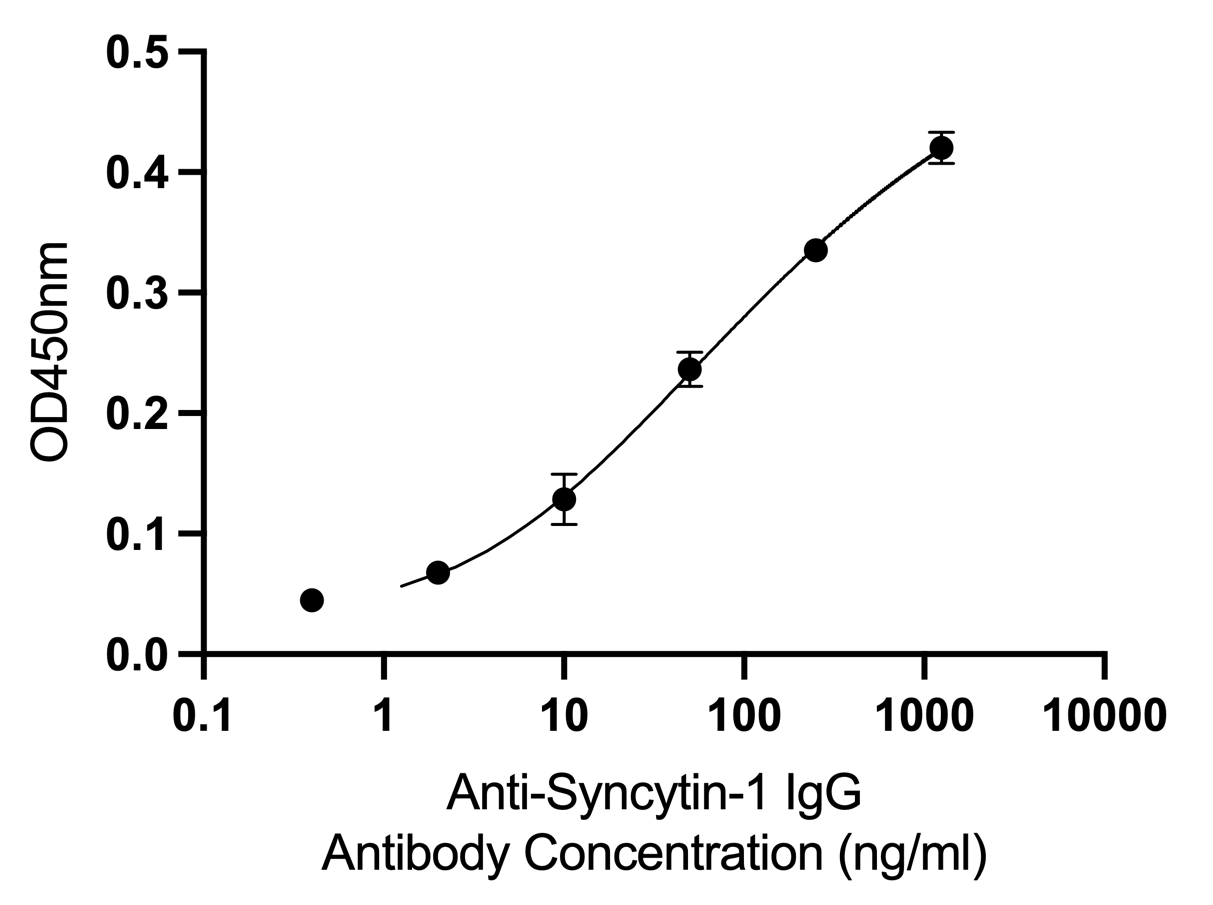

Supplement: S1 Fig — Mean absorbance (OD450nm) plotted against serial dilutions of monoclonal IgG antibody against syncytin-1 to generate a standard curve. Best fit was determined using asymmetrical sigmoidal 5-parameter least-squares fit. Projected antibody concentrations were interpolated using this fit. Mean and standard deviation of representative data is shown. R-squared = 0.9996, Sum of squares = 6.572 × 10^−5^. The underlying source data for this figure can be found in S1 Data. HERV-W, human endogenous retrovirus W; IgG, immunoglobulin G. (TIFF) [file pbio.3001506.s001.tiff]

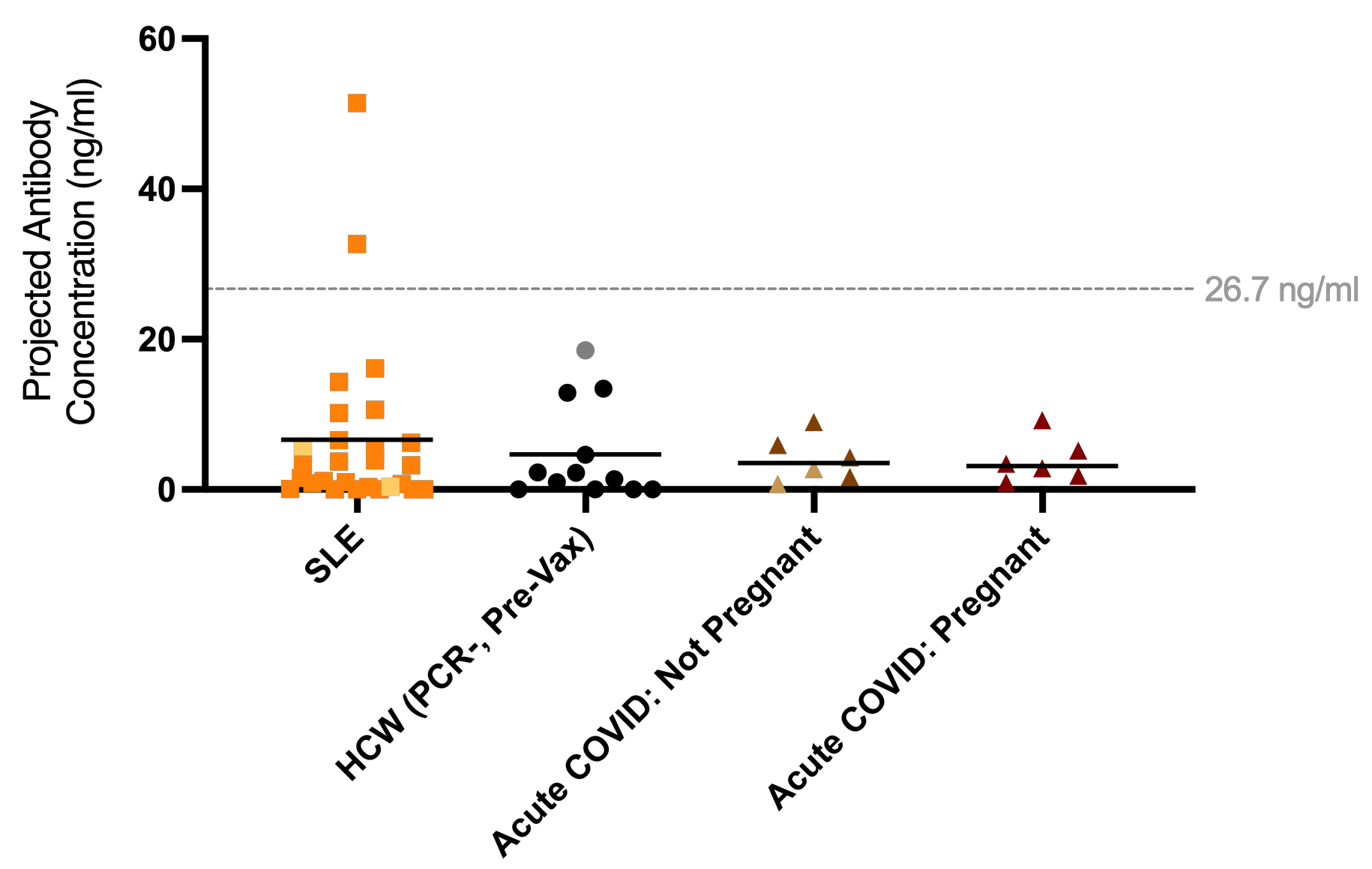

Supplement: S2 Fig — Plasma reactivity to syncytin-1 protein was assessed by ELISA in SLE samples (n = 27), unvaccinated HCW samples (n = 12), nonpregnant patients with acute COVID-19 disease (n = 6), and pregnant patients with acute COVID-19 disease (n = 6). Each dot represents a single individual. Male participants are lightened in color. Horizontal bars represent mean values. Statistical significance was assessed using nonparametric Mann–Whitney tests. No groups were significantly elevated as compared to HCW controls. Horizontal dashed line (drawn at 26.7 ng/ml) represents maximum of kernel distribution estimate for HCW (PCR-, Pre-Vax) control samples, indicating the upper limit of the normal range in healthy, unvaccinated individuals. The underlying source data for this figure can be found in S1 Data. COVID-19, Coronavirus Disease 2019; HCW, healthcare worker; HERV-W, human endogenous retrovirus W; SLE, systemic lupus erythematosus. (TIFF) [file pbio.3001506.s002.tiff]
