# Supplementary material for: No evidence of fetal defects or anti-syncytin-1 antibody induction following COVID-19 mRNA vaccination
Source: PLoS Biol. 2022 May 24;20(5):e3001506. doi: 10.1371/journal.pbio.3001506 (PMC9129011; doi:10.1371/journal.pbio.3001506)
Supplement: Attachment — Submitted filename: 2022.03.07_COVIDvaxpreg_ResponseToReviewers.docx [file pbio.3001506.s004.docx]

We thank the reviewers for their careful review of this manuscript, and for the insightful comments and criticism. In our revised submission, we have addressed all the points raised by reviewers and clarified important details of our study as requested.

Notably, we have dramatically increased the sample size and power of our study of anti-syncytin-1 antibody levels. In our revision, we include a new independent cohort of matched samples from 41 individuals and have added more samples to our control (6 additional uninfected, unvaccinated healthcare worker samples) and comparison groups (11 additional SLE patient samples) as well. Working with a statistician, we used these expanded datasets to model estimates of the probability of increased syncytin-1 levels in unvaccinated vs. vaccinated groups. These analyses show that vaccinated groups do not have a higher probability of having anti-syncytin-1 antibody levels above a normal range compared to healthy, unvaccinated healthcare worker controls. Finally, we have added new references and clarified the methods to more clearly convey the techniques used for fetal serum collection and antibody measurement.

We hope that you will agree that in addressing each of the reviewers’ comments and by providing the additional information requested, we have significantly strengthened the manuscript.

**Response to reviewers:**

Reviewer #1: This is an important manuscript that experimentally addresses two common

concerns around COVID vaccination in pregnancy. The inclusion of SLE patients to demonstrate

that the author's assay for the detection of anti-syncytin1 antibodies can detect them when

they are present is a particular strength. Prior to publication I suggest that the authors address

the following points.

Major revision

I could not find any methodological information about the ELISA used to produce the data

shown in Figure 2.

We thank the reviewer for raising this important point. We now recognize that the methodological information about the ELISAs used to produce the data in Figure 2 were not clearly stated and in part buried in the section describing the measurement of human syncytin-1 antibodies. We apologize for this error and have added a Methods subsection (“*SARS-CoV-2-specific antibody measurements*”) more clearly stating the details of the techniques used.

The reason I was looking for this information is that the result presented is

unexpected. Unlike humans, rodents do not undergo significant transplacental IgG transfer;

rather antibody transfer occurs mainly in the neonatal period, via milk and a "leaky" gut (see for

example Pentsuk, 2009, Birth Defects Res B Dev Reprod Toxicol). But since these pups were

collected at e18.5, they cannot have received this antibody via milk. Therefore, I was looking for

details of exactly what was measured and how to work out if there might be some technical

explanation for this surprising result. The authors should discuss their methods, and the

unexpected result here in more detail. Alternatively, given that we do not consider the mouse to

be a particularly good model for transplacental antibody transfer as seen in humans, the

authors may choose not to show this result.

The reviewer is correct that the pups were collected prior to birth at E18.5 and thus cannot have received antibodies via milk. This finding is consistent with a large body of literature demonstrating that in utero maternal-fetal antibody transfer does indeed occur in mice and can play an major role in fetal and neonatal outcomes.

In regard to the presence of antibodies in E18.5 pups, the review article by Pentsuk et al. 2009 summarizes studies that suggest that most IgG transmission in rats occurs after birth, but this is not extended to mouse models. Regarding mouse models, Pentsuk et al. 2009 simply state in this paper, “No reports were identified in which the maternofetal transfer of mouse IgG has been studied.” Notably, this statement is not accurate because there are a number of studies both prior to and following the time of this review that clearly demonstrate that antibodies are transferred in utero from the pregnant dam to the fetus in mouse models[1-12]. This includes in established models of viral infection[1,4,5,9], as well as mouse gestational transfer models that demonstrate that even human antibodies injected into pregnant dams are similarly able to cross the murine maternal-fetal interface[3,6,8,10,11]. Other work has explored the mechanism of this transplacental IgG transfer in mice (including at the same gestational stages that we use in this manuscript), showing that efficient transfer is dependent on FcRn expression[7,12].

Our study thus adds to the growing body of evidence showing that rodent models do exhibit robust transplacental antibody transfer during pregnancy. We show from our work that maternal-fetal antibody transfer clearly does occur prior to birth. Circulating antibodies in the fetus are sustained at term even when maternal vaccination takes place at timepoints preceding the formation of the definitive placenta, with fetal levels of SARS-CoV-2 Spike antibody reaching levels approaching that in the maternal circulation as clearly shown in Figure 2.

We thus choose to present these results as stated, but in order to add clarity in response to the reviewer’s comments, we have added more direct language to the Methods section (in “*Harvest of fetuses, fetal serum collection, and fetal measurements*”) detailing the intact recovery of E18.5 fetal bodies, the absence of any outside contamination with maternal blood during dissection, and the direct collection of blood from cleanly isolated and rinsed decapitated fetal bodies.

Minor points.

Introduction. Discussion of references 9-11 on vaccine safety. It is worth noting that Zauche is a

follow up on Shimabukuro, also in the V-safe cohort. It seems strange to note the limitated

follow-up time in Shimabukuro, when Zauche, who did the requisite follow-up in the same

cohort, is cited in the very next sentence. I would also cite Kharbanda, 2021, NEJM, which uses

Vaccine Safety Datalink data - then all the major studies on this from the USA will have been

cited. There are others, from other countries, but they all show much the same thing and I

appreciate that this is not a literature review.

Thank you for bringing up this suggestion regarding our referencing. We agree with the reviewer and have edited our references to align with the reviewer’s excellent suggestion.

References 21 and 22 are given to say there is no evidence to support claims that vaccines harm fertility. For completeness, the authors might also wish to cite Morris, 2021, F&S Reports;

Orvieto, 2021, Reprod Biol Endocrinol; Safrai, 2021, MedRXiv.

Discussion. The authors mention that Mattar, 2021, MedRXiv has already shown that

vaccination does not raise antibodies to syncytin1 (although their assay was rather less

convincing than the one presented here, in my view). They may also wish to cite Prasad, 2021,

Cell Mol Immunol, which also shows this.

We again thank the reviewer for their superb suggestions on the inclusion of additional references in this rapidly developing field. The references provided are directly relevant and have been included in the revised manuscript. We have also included additional references that have been emerged since our submission that are directly relevant to our work.

Reviewer #2: This manuscript by Lu-Culligan and colleagues report an investigation into the

impact of covid-19 mRNA vaccination on fetal development and anti-syncytin antibody

induction that might impact placental function. The authors combined the use of a mouse

model and anti-syncytin antibody measurements in plasma samples obtained from covid-19

vaccinated individuals. They showed that mRNA-1273 vaccination not only did not produce any

observable effect on fetal development in mice, it also produced anti-spike antibodies that were

transferred vertically across the placenta to the fetuses. They also showed that that mRNA

covid-19 vaccination did not produce any detectable anti-syncytin antibodies and thus

concluded that their findings contradict popular claims of safety concerns in vaccinating

pregnant women.

The topic of this study is timely and much needed to produce evidence to combat widespread

misinformation on the safety of mRNA vaccines. The main challenge in such a study is that the

conclusion is based entirely on negative findings. There are thus several important questions

that still need to be considered to fully support the conclusion.

1. Unlike drugs, vaccine dose is not determined based on body weight. What the authors have

used is 2x the dose used in pre-clinical demonstration of safety and efficacy of this same

vaccine. Conceivably, the authors have used more vaccine than what is necessary for

immunogenicity. However, it still does not directly address the issue on whether 100 ug is safe

in pregnant women.

We thank the reviewer for their comments and the opportunity to address questions surrounding the dosage used. We recognize that vaccine dosing in humans is not determined by body weight and provide only rough vaccine dosage by body weight used in the animal studies to serve as a comparison point since mice were not individually weighed and dosed.

Given that the primary aim of the study was to establish safety of the vaccine in pregnancy and early fetal development, we chose to use a vaccine dosage over 50 times the amount administered to humans with the rationale that any potential detrimental effects would be mostly likely be revealed and most pronounced at this much higher range of exposure. As the reviewer states, such an outsized dose would be expected to exhibit increased immunogenicity at these levels of treatment, allowing increased sensitivity for our study to detect any revealed defects in development.

Notably, the commercial formulation of the mRNA vaccines also sets limitations as to the amount that can be experimentally administered to mice without introducing additional variables. We chose to keep the vaccine formation intact at the manufactured concentration without diluting or concentrating any of the precisely formulated components of the vaccine, mRNA or otherwise, in our experiments. Administration of the human dose of vaccine 100ug (500 ul) into mouse muscle is thus not only technically and ethically unfeasible, but we believe entirely unnecessary in this model.

In summary, we chose to keep the vaccine formulations intact and administer a large dose to animal subjects because the primary goal of this study is to demonstrate safety of the vaccine and its components during pregnancy. However, we do not feel it is necessary to administer human levels of vaccine into pregnant mice in order to demonstrate its safety.

**As a positive control, the authors had used 50 ug of poly IC to show impact**

**of over-activation of RNA sensors on fetal development. Perhaps the authors would consider an**

**"immunobridging" experiment in non-pregnant mice by comparing different doses of**

**mRNA-1273, preferably up to 100 ug with 50 ug poly IC, to show that the chemically modified**

**nature of mRNA-1273 to evade RNA sensors would avoid the level of inflammation needed to**

**impact normal fetal development?**

The second point brought up regarding the comparison of poly(I:C) to vaccine mRNA is also valid but is limited by technical constraints.

Again, the experiment proposed by the reviewer in which mice are vaccinated with up to 100ug of mRNA-1273 is a great suggestion in theory, but in practicality cannot be performed in this case, at least not without significantly altering the constitution of the vaccine. The volume needed to inject would be 500 ul, into the muscle of the mouse, which is both ineffective due to tissue disruption and would also not be approved due to clear ethical concerns. For such volumes to be used, the injection would have to be administered by intravascular or intraperitoneal injection, thus not mimicking the vaccine route used in humans.

We used a maximum of 50 ul for all intramuscular injections in our experiments to observe the upper limit of a safe injectable volume. According to Gehling et al., intramuscular volumes greater than 50 ul injected into the caudal thigh of mice (the technique used in this study) lead to leakage into extramuscular tissues, with volumes over 200 ul resulting in moderate to severe leakage[13]. Furthermore, we again feel that the use of a 100 ug (500 ul) comparison is not necessary as we already provide data at a dosage over 50 times the amount by body weight that is administered in humans.

Finally, the “immunobridging” experiment proposed is not feasible because it is unknown what the appropriate read-out or endpoint comparison should be used to compare poly(I:C) with mRNA-1273. While the inflammatory mediators impacting normal fetal development can be hypothesized, they remain uncharacterized and it is unclear what key players, responses, or cytokines/factors are involved, let alone the tissue or organ localization of the most relevant responses.

For example, when immunobridging studies have been used to compare COVID-19 vaccines, they are able to measure a common endpoint of antibody response levels to SARS-CoV-2. By contrast, in the case of immune activation by poly(I:C) administration, it remains unknown what exact pathway leads to decreased fetal size and weight, and it is unlikely to be traced to a single mediator. There are a constellation of potential inflammatory markers and changes associated with poly(I:C) treatment to consider; we therefore simply lack clarity on the direct readout required to perform such an immunobridging comparison study.

In the manuscript, we propose that TLR3-mediated immune responses may explain the differences in phenotype but this remains a hypothesis and remains underexplored in the literature. We also believe this is beyond the scope of this paper which simply seeks to link establish in this mouse model that maternal vaccination with mRNA-1273 does not impact fetal growth in utero.

2. The demonstration of vertical transfer of antibodies from dams to fetuses is less useful now

that there is human data.

We recognize that there are indeed studies with human data showing vertical transfer of antibodies, but these studies analyzed women vaccinated in late pregnancy, while our animal model provides evidence of robust antibody transfer at term even after vaccination in early pregnancy, prior to the formation of the definitive placenta and analogous to the human first trimester.

Due to the limited numbers of women receiving the vaccine in the first trimester pre-placentation (before or at the typical time of the first ultrasound), the human data on vertical antibody transfer are understandably limited by the same skew seen in studies of safety. For example, the cord blood study by Mithal et al. 2021 reports an average gestational age at first vaccine dose of 33±2 weeks, well into the third trimester[14]. Our animal model provides evidence that robust transplacental transfer of antibodies is sustained over the timespan of pregnancy, even when vaccines are administered before the placenta has developed.

Additionally, our results demonstrate vertical antibody transfer prior to parturition or transfer of antibodies via milk (see our discussion of Reviewer #1’s comments above). This observation further informs the future use of animal models for vaccination and vertical transfer during pregnancy.

3. The demonstration of lack of anti-syncytin antibodies is very much limited by the small

sample size. Perhaps the authors would consider working with a statistician to estimate the

level of risk of development of anti-syncytin antibodies that could be excluded by this study? It

is unclear from the methods if random sampling was applied to select the plasma samples for

analysis. If not, perhaps the statistical consideration could be used in conjunction with random

sampling of another batch of plasma/serum samples from vaccinated individuals?

In response to the reviewer’s comments, we have dramatically increased our sample size by adding a new independent cohort of 41 samples to our dataset. This new cohort from the Dominican Republic is a random sampling of another population that received CoronaVac (two doses of inactivated whole-virion vaccine) and were then boosted with the BNT162b2 mRNA vaccine. The results from this second cohort are consistent with the previous cohort described, with no induction of anti-syncytin-1 antibodies observed when comparing matched samples pre-booster, 7 days post-booster, and 28 days post-booster with the BNT162b2 mRNA vaccine. Given the two independent cohorts now included in Figure 3, we have directly addressed the reviewer’s concerns and added another random sampling as suggested. Together these findings represent the largest study of anti-syncytin-1 antibody status in individuals receiving the COVID-19 mRNA vaccines to date.

We have also added more samples to the control and comparison groups. In this revision, we have included 11 more SLE patient samples and 6 more unvaccinated, uninfected healthcare worker controls.

Finally, as suggested by the reviewer, we worked with a statistician, Dr. Shuangge Ma, to model an estimate of the level of risk that individuals in these vaccination groups may be to having abnormal anti-syncytin-1 levels.

Dr. Ma has been added as a co-author and his analysis is described as follows.

For each column in the excel file, we fit a parametric distribution. With the small sample sizes, we choose simple models. In particular, we choose the exponential distribution for all columns except for pre-vax, to accommodate the excessive small values close/equal to zero. For pre-vax, we fit a Gamma distribution. We do acknowledge the limitation of these simple models. However, with the limited sample sizes, more complicated models may not be reliable. The histograms and fitted models are shown below:

Then based on the fitted models, we calculate the probabilities of beyond 28.8 – the probabilities of being out of the normal range.

Table 1: Summary statistics: mean, standard deviation (SD), estimated parameters ($\lambda,\alpha,\beta$), and probability of exceeding 28.8.

|  | Mean | SD | $\lambda\backslash\alpha$ | $\beta$ | $P(X>28.8)$ |
| --- | --- | --- | --- | --- | --- |
| HCW (Pre-Vax) | 5.1175 | 43.5836 | 0.1954 |  | 0.0036 |
| SLE | 6.6390 | 130.8891 | 0.1506 |  | 0.0131 |
| Cohort 1: Pre-Vax | 3.6482 | 6.1736 | 2.1559 | 0.5909 | 0.0000 |
| Cohort 1: Post-Vax (7 days post dose 2) | 4.4339 | 17.9425 | 0.2255 |  | 0.0015 |
| Cohort 1: Post-Vax (28 days post dose 2) | 5.0270 | 22.3278 | 0.1989 |  | 0.0033 |
| Cohort 2: Pre-mRNA Vax | 2.7298 | 13.6132 | 0.3663 |  | 0.0000 |
| Cohort 2: Post- mRNA Vax (7 days) | 3.9645 | 25.5141 | 0.2522 |  | 0.0007 |
| Cohort 2: Post- mRNA Vax (28 days) | 3.1650 | 24.6049 | 0.3160 |  | 0.0001 |

As demonstrated above, the estimated probability of an abnormal range of anti-syncytin-1 antibodies, P (X > 28.8), is exceedingly low for all vaccinated groups, both Cohort 1 and Cohort 2.

None of the probabilities for any vaccinated group at any vaccinated time point exceeds that of our control healthy, unvaccinated population, HCW (Pre-Vax).

Any additional questions or clarifications on the statistical model used can be directed to Dr. Shuangge Ma at [shuangge.ma@yale.edu](mailto:shuangge.ma@yale.edu).

Reviewer #3: In this brief manuscript, Culligan et al study two aspects of COVID-19 in pregnant

women. First, the effect of vaccination on fetal growth in mice was assessed. Second, the

authors measured levels of anti-syncytin-1 antibody in pregnant women. While the results are

reassuring and support the recommendations for the use of mRNA vaccines in pregnancy, the

mouse study is underdeveloped and the number of patients studied is very small. Also, the two parts of the manuscript are not well connected.

Specific comments.

1. The dose of mRNA vaccine used in mice is high relative to the human dose, but is the level

excessively high in its ability to induce a protective immune response?

We thank the reviewer for this question. We demonstrate that the increased dose of the mRNA vaccine leads to robust anti-SARS-CoV-2 Spike antibody levels following administration and that these levels are maintained over weeks of murine pregnancy to term in both the pregnant dam and fetuses in utero. Thus, we show that the level is not excessively high in its ability to induce a protective immune response.

We also have data from mice treated with higher and lower levels of vaccine, which show that the antibody levels that result do not significantly differ based on the range of vaccine we used.

2. An additional concern of those pregnant women who are vaccine skeptic is whether vaccine

mRNA crosses the placenta. This should be possible to assess in mice.

We agree that this is an interesting question and one that may be more directly explored in the future but this aim is beyond the intended scope of this paper. We cite two studies in the manuscript that did not identify IgM antibodies in any neonate studied[14,15], suggesting that the mRNA in the vaccine is unable to cross the placenta to trigger an endogenous antibody response in the infant. While this is an indirect analysis, these studies have been cited as evidence that mRNA is not crossing the placenta following maternal vaccination.

3. The human studies are important, but as the authors point out, the numbers are too small to

detect an elevated syncytin-1 antibody response in even a substantial minority of patients.

The extremely low prevalence of elevated anti-syncytin-1 antibodies in the population is expected and not a limitation of the study. Our aim in the manuscript is to address misinformation claiming that anti-syncytin-1 levels surge to high levels post-vaccination and we show that they do not. The fact that the starting prevalence is so low does not impact the strength of our conclusions because we are analyzing the change in anti-syncytin-1 levels in response to vaccination, which we convincingly show does not increase from minimal starting levels and from healthy, unvaccinated control populations (see previously stated statistical analysis and Table 1 above).

We do not claim that elevated anti-syncytin-1 levels are common in either the general population or SLE patients more likely to exhibit autoreactive antibody profiles. Instead, we demonstrate that this is an exceedingly rare phenotype, whether pre- or post-vaccination, lending further support to the implausibility of the anti-syncytin-1 misinformation.

Finally, as repeated from above, we have increased the number of samples analyzed using a new cohort of patients described in the revised manuscript. We added anti-syncytin-1 antibody data for 41 human participants that had received CoronaVac (two doses of inactivated whole-virion vaccine) before and after they were boosted with the BNT162b2 mRNA vaccine. We have also added 11 SLE patient samples and 6 unvaccinated, uninfected healthcare worker controls to increase our power. These findings represent the largest dataset available on anti-syncytin-1 status in individuals receiving the COVID-19 mRNA vaccine and, with the statistical analysis provided above, we show that relative probabilities of elevated syncytin-1 remain low compared to SLE and healthy controls both before and after vaccination of two independent randomly sampled cohorts.

References

1.	Hayashi Y, Wada T, Mori R. Protection of newborn mice against herpes simplex virus infection by prenatal and postnatal transmission of antibody. J Gen Virol. 1983;64: 1007–1012. doi:10.1099/0022-1317-64-5-1007

2.	Lazarova Z, Yee C, Darling T, Briggaman RA, Yancey KB. Passive transfer of anti-laminin 5 antibodies induces subepidermal blisters in neonatal mice. J Clin Invest. American Society for Clinical Investigation; 1996;98: 1509–1518. doi:10.1172/JCI118942

3.	Tran HB, Macardle PJ, Hiscock J, Cavill D, Bradley J, Buyon JP, et al. Anti-La/SSB antibodies transported across the placenta bind apoptotic cells in fetal organs targeted in neonatal lupus. Arthritis Rheum. 2002;46: 1572–1579. doi:10.1002/art.10316

4.	Yorty JL, Bonneau RH. Transplacental transfer and subsequent neonate utilization of herpes simplex virus-specific immunity are resilient to acute maternal stress. Journal of Virology. 2003;77: 6613–6619. doi:10.1128/jvi.77.12.6613-6619.2003

5.	Yorty JL, Bonneau RH. Prenatal transfer of low amounts of herpes simplex virus (HSV)-specific antibody protects newborn mice against HSV infection during acute maternal stress. Brain Behavior and Immunity. 2004;18: 15–23. doi:10.1016/s0889-1591(03)00115-6

6.	Singer HS, Morris C, Gause C, Pollard M, Zimmerman AW, Pletnikov M. Prenatal exposure to antibodies from mothers of children with autism produces neurobehavioral alterations: A pregnant dam mouse model. Journal of Neuroimmunology. Elsevier B.V; 2009;211: 39–48. doi:10.1016/j.jneuroim.2009.03.011

7.	Kim J, Mohanty S, Ganesan LP, Hua K, Jarjoura D, Hayton WL, et al. FcRn in the yolk sac endoderm of mouse is required for IgG transport to fetus. J Immunol. 2009;182: 2583–2589. doi:10.4049/jimmunol.0803247

8.	Braunschweig D, Golub MS, Koenig CM, Qi L, Pessah IN, Van de Water J, et al. Maternal autism-associated IgG antibodies delay development and produce anxiety in a mouse gestational transfer model. Journal of Neuroimmunology. 2012;252: 56–65. doi:10.1016/j.jneuroim.2012.08.002

9.	Fowler AM, Tang WW, Young MP, Mamidi A, Viramontes KM, McCauley MD, et al. Maternally Acquired Zika Antibodies Enhance Dengue Disease Severity in Mice. Cell Host and Microbe. 2018;24: 743–750.e5. doi:10.1016/j.chom.2018.09.015

10.	García-Serra A, Radosevic M, Pupak A, Brito V, Ríos J, Aguilar E, et al. Placental transfer of NMDAR antibodies causes reversible alterations in mice. Neurol Neuroimmunol Neuroinflamm. 2021;8. doi:10.1212/NXI.0000000000000915

11.	García-Serra A, Radosevic M, Ríos J, Aguilar E, Maudes E, Landa J, et al. Blocking Placental Class G Immunoglobulin Transfer Prevents NMDA Receptor Antibody Effects in Newborn Mice. Neurol Neuroimmunol Neuroinflamm. Wolters Kluwer Health, Inc. on behalf of the American Academy of Neurology; 2021;8. doi:10.1212/NXI.0000000000001061

12.	Coutinho E, Jacobson L, Shock A, Smith B, Vernon A, Vincent A. Inhibition of Maternal-to-Fetal Transfer of IgG Antibodies by FcRn Blockade in a Mouse Model of Arthrogryposis Multiplex Congenita. Neurol Neuroimmunol Neuroinflamm. Wolters Kluwer Health, Inc. on behalf of the American Academy of Neurology; 2021;8. doi:10.1212/NXI.0000000000001011

13.	Gehling AM, Kuszpit K, Bailey EJ, Allen-Worthington KH, Fetterer DP, Rico PJ, et al. Evaluation of Volume of Intramuscular Injection into the Caudal Thigh Muscles of Female and Male BALB/c Mice (Mus musculus). J Am Assoc Lab Anim Sci. 2018;57: 35–43.

14.	Mithal LB, Otero S, Shanes ED, Goldstein JA, Miller ES. Cord blood antibodies following maternal coronavirus disease 2019 vaccination during pregnancy. American Journal of Obstetrics and Gynecology. 2021;225: 192–194. doi:10.1016/j.ajog.2021.03.035

15.	Beharier O, Plitman Mayo R, Raz T, Nahum Sacks K, Schreiber L, Suissa-Cohen Y, et al. Efficient maternal to neonatal transfer of antibodies against SARS-CoV-2 and BNT162b2 mRNA COVID-19 vaccine. J Clin Invest. 2021;131. doi:10.1172/JCI150319
